# Fish Utilisation of Wetland Nurseries with Complex Hydrological Connectivity

**DOI:** 10.1371/journal.pone.0049107

**Published:** 2012-11-09

**Authors:** Ben Davis, Ross Johnston, Ronald Baker, Marcus Sheaves

**Affiliations:** Estuary and Tidal Wetland Ecosystems, School of Marine and Tropical Biology, James Cook University, Townsville, Queensland, Australia; Institute of Marine Research, Norway

## Abstract

The physical and faunal characteristics of coastal wetlands are driven by dynamics of hydrological connectivity to adjacent habitats. Wetlands on estuary floodplains are particularly dynamic, driven by a complex interplay of tidal marine connections and seasonal freshwater flooding, often with unknown consequences for fish using these habitats. To understand the patterns and subsequent processes driving fish assemblage structure in such wetlands, we examined the nature and diversity of temporal utilisation patterns at a species or genus level over three annual cycles in a tropical Australian estuarine wetland system. Four general patterns of utilisation were apparent based on CPUE and size-structure dynamics: (i) classic nursery utlisation (use by recently settled recruits for their first year) (ii) interrupted peristence (iii) delayed recruitment (iv) facultative wetland residence. Despite the small self-recruiting ‘facultative wetland resident’ group, wetland occupancy seems largely driven by connectivity to the subtidal estuary channel. Variable connection regimes (i.e. frequency and timing of connections) within and between different wetland units (e.g. individual pools, lagoons, swamps) will therefore interact with the diversity of species recruitment schedules to generate variable wetland assemblages in time and space. In addition, the assemblage structure is heavily modified by freshwater flow, through simultaneously curtailing persistence of the ’interrupted persistence’ group, establishing connectivity for freshwater spawned members of both the ‘facultative wetland resident’ and ‘delayed recruitment group’, and apparently mediating use of intermediate nursery habitats for marine-spawned members of the ‘delayed recruitment’ group. The diversity of utilisation pattern and the complexity of associated drivers means assemblage compositions, and therefore ecosystem functioning, is likely to vary among years depending on variations in hydrological connectivity. Consequently, there is a need to incorporate this diversity into understandings of habitat function, conservation and management.

## Introduction

Increasing knowledge of temporal utilisation patterns of functional groups, and of the underlying processes regulating their occurrence has led to great advances in our understanding of the functioning of estuarine fish assemblages [1]. Such studies have primarily concerned subtidal estuary channels (hereafter referred to simply as ‘estuary channels’), however the coastal and estuarine system acts as a mosaic of inter-connected habitats, linked through fish migrations at a range of scales, including feeding and refuge, ontogenetic, and life-history migrations [2]. Consequently, complete understanding of estuarine function will not be achieved without understanding the utilisation of other important estuarine habitats [3].

Occurring adjacent to estuary channels worldwide are a variety of fringing wetlands with varying potential for fish utilisation. Vegetated intertidal wetlands (i.e. mangrove forests and saltmarshes) are prominent and iconic components of estuarine systems, and provide tidally available habitat for fauna inhabiting the estuary channel [4]. Periodic tidal emersion means that temporal utilisation patterns are a function of seasonal dynamics in the estuary channel, modified by tidal-driven migration patterns [5]. Estuarine systems worldwide also contain a variety of floodplain wetlands, comprising a mixture of pools, lakes, lagoons and seasonally flooded lowlands, which occupy a range of settings (e.g. saltpan, pasture, saltmarsh) and connect to the estuary channel over a range of temporal and spatial scales. Although estuarine floodplain wetlands are recognised as important nurseries for fish [6], [7], detailed knowledge of utilisation patterns is scant. Floodplain wetlands provide relatively permanent habitats (often persisting through tidal and annual cycles) which nekton potentially use for longer periods, spanning tidal visits to periods of years, depending on wetland persistence, and the frequency and duration of hydrological connection to the estuary channel. Consequently, floodplain wetlands provide alternative habitats to the estuary channel, providing the possibility of separate nursery function, and different patterns of occupation.

The dynamic regimes of hydrological connectivity characteristic of estuarine floodplain wetlands, featuring the interplay of tidal marine and freshwater connections, results in variable physical conditions, and simultaneously provides corridors for fish recruitment from both estuarine and freshwater systems [8]. In dry- and sub-tropical wetlands, patterns of hydrological connectivity and resulting physical dynamics are particularly pronounced. Periods of low or negligible rainfall generally extend through much of the year often resulting in floodplain wetlands drying to a fragmented series of tidally connected pools (hereafter termed ‘estuarine pools’) [9] with an increased propensity to become hypersaline due to dislocation from freshwater reaches and reduced tidal connectivity [10]. A discrete wet season characterised by increased freshwater flows (∼January-March) can then induce abrupt and severe drops in salinity, and shifts in other physical parameters [11], while establishing or enhancing connections to both freshwater and estuarine sources [8]. Conditions then become increasingly saline through the rest of the year as freshwater is progressively replaced by coastal marine water [10]. The consequences of these changes for fish utilisation patterns are poorly understood, however these dynamics are likely to interact with variable physiological tolerances of organisms to modify patterns of wetland utilisation for many species [12], [13].

Estuarine pools have received some attention in Australia’s tropics [14], [15] and sub-tropics [8], [16], with a focus on the nursery function for the commercially and recreationally important barramundi, *Lates calcarifer*. *L. calcarifer* spawn in coastal waters and mouths of estuaries during wet season months, coinciding with periods when connectivity and habitat availability of fragmented coastal wetlands is greatest [17]. Consequently, juvenile barramundi recruit to estuarine pools during wet season months [18]. They remain until the advent of the dry season, although it is unclear whether this represents a life-history emigration or if occupancy is curtailed by declining water levels or water quality. Despite the past focus on *L. calcarifer,* wetland fish fauna are taxonomically diverse [9], [14], [15]. Components of estuarine wetland assemblages show a variety of spawning schedules [19], [20] and life-history strategies [21], which together with variable physical tolerances mean assemblages are likely to display a diversity of pool occupation patterns, featuring modified timing and age of recruitment, and subsequent persistence of different species.

To develop an understanding of the patterns and underlying processes driving the fish assembage of estuarine pools in the tropics, we examined the nature and diversity of temporal utilisation patterns (timing and age at recruitment, and subsequent persistence) at a species level over three annual cycles that incorporated strong physical change. The study focused on a natural wetland system comprised of 20 discrete pools situated on a salt-marsh of the Ross River in North Queensland, Australia. Available data available from the main channels of estuaries in the region were used to provide a utilisation pattern ‘null model’, to investigate whether observed patterns were typical of estuary channel use, or if different sets of processes influenced floodplain wetland utilisation.

## Methods

### Ethics Statement

This study was undertaken with the approval of James Cook University Animal Ethics Committee (permit A1466), in accordance with institutional, national, and international animal ethics guidelines, and under Queensland fisheries permit requirements (permit number: 114026). This study did not involve any endangered or protected species. No specific permits were required to access or conduct work on the field site, since the land is not protected nor privately owned.

### Study Site

**Figure 1:**
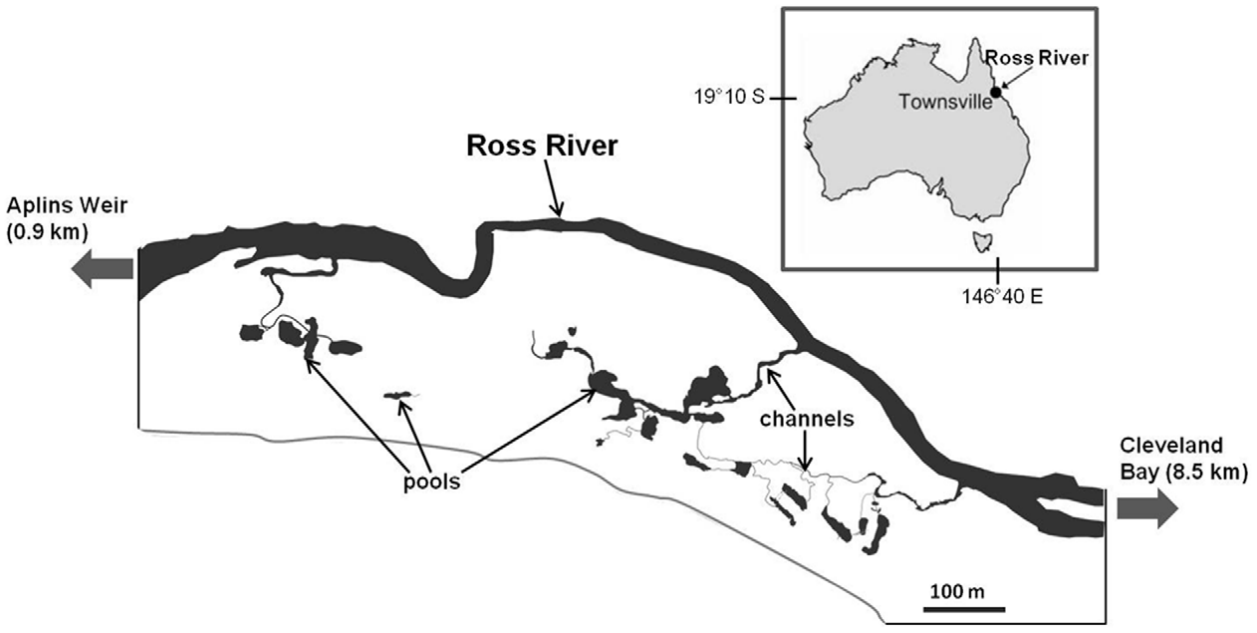
Geographic location of Annandale Wetland and map of the 20 pools on the wetland.

The study was conducted in Annandale Wetland (19.19°S; 146.44°E) (Fig. 1), a 0.4 km^2^ *Sporobolous virginicus* salt-marsh system situated on the Ross River floodplain, 8 km’s upstream of the river mouth. Interspersed across the wetland are 20 discrete permanent pools, ranging in area from 80 m^2^–2500 m^2^, and in low-tide depth from 30 cm–130 cm. The pools are mostly unvegetated and generally lack permanent complex structure. They encompass a range of substrates, varying from fine mud to coarse rubble.

Weather patterns in the study region can be divided into 4 periods [20]: (1) a pronounced hot wet season, generally concentrated around January-March, yet occasionally extending into neighbouring months. During years where there is sufficient rainfall Aplin’s Weir (located 0.9 km upstream) overflows, blanketing the wetland in a sheet of freshwater. (2) A post-wet season (∼April-May), where conditions begin to cool. During this transition period floodwaters naturally recede (hereafter referred to as draw-down), and the system of pools are revealed as discrete semi-isolated units connected to each other and to the Ross River to varying extents during high tides. The majority of pools are connected via narrow channels (ranging in width from ∼0.5 m to 10 m) during most lunar tidal cycles, although a minority rely on spring tides to receive connection over the salt-marsh surface. This state of alternating marine connections and disconnections continues though (3) a cool dry season (June-September); and (4) a pre-wet season (October-December) where conditions begin to warm prior to the commencement of the wet season.

### Fish Sampling

Sampling of all 20 pools commenced after wet season floodwaters receded in March 2010, and was repeated on a monthly basis until the commencement of the following wet season in December. Monthly sampling in the first three months following draw-down was undertaken during 2011, to incorporate likely complexity associated with this transition period, followed by bi-monthly sampling until the end of the year. A third annual sample was collected for the first month after floods in April 2012.

All samples were collected over the bottom quarter of the tidal cycle during the new moon phase to ensure consistent tidal regimes throughout the study. Sampling was conducted using a seine net (12 m long, 2 m deep, 6 mm mesh), with an effective sampling width of 8 m. Some pools could be comprehensively sampled in a single seine haul. However, larger pools required up to 3 separate hauls to include the range of habitats present. Complete coverage of habitats was needed to fully represent each taxa in each pool, controlling for any within-pool microhabitat associations. Fish numbers and their sizes were recorded (measured in 10 mm fork length (FL) size classes, and reported as size-class minimums (e.g. 27 mm FL = 20 mm)). Fish <10 mm FL were excluded from analyses as a large proportion were below mesh selection size, and unlikely to be well represented. The catch was returned to the water alive, with the exception of the noxious pest species, *Oreochromis mossambicus*, which was euthanized on site in accordance with fisheries requirements. Minimising mortalities through the rapid return of catch was integral to the study, as extraction may substantially influence catch in subsequent months. Salinity, water temperature, and visibility (Secchi depth) were recorded in each pool on each sampling occasion as potential explanatory variables of fish dynamics. Freshwater flow data over Aplin’s Weir were also provided by North Queensland Water.

Data collected across the complete lengths of 9 small North Queensland estuary channels as part of a previous study [20] were interrogated to develop a null model of expected wetland utilisation patterns. Many species of fish using estuarine pools are widespread across the region’s coastal and estuarine system [22], and any difference in utilisation pattern between the wetland and the estuary channel null model provided an insight into processes shaping wetland utilisation. For instance, it enabled assessment of whether any disparity in utilisation pattern was a function of different regimes and severities of physical change between the two habitats. Fish were quantitatively sampled from the estuary channels using cast nets (5 mm mesh size) during 12 sampling trips between November 2007 and January 2009; the complete methodology can be found in Sheaves et al. (2010) [20]. Since cast nets and seine nets are both effective at sampling the main components of the small fish assemblage in tropical estuaries [23], general comparisons of temporal population dynamics (from which utilisation patterns could be interpreted) could be made for well represented taxa. To standardise the range of analysed size classes with the Annandale Wetland seine data, fish <10 mm FL were also excluded. Data were not available for the Ross River channel, and sampling the channel in addition to the pools was beyond the scope of the present study. However, the objective was not to make a direct comparison between pool and channel habitat within a system, but rather to assess whether patterns in Annandale Wetland reflected general patterns of estuary use.

### Data Analysis

The most commonly captured species’ were selected for analysis, along with some larger less-abundant species of commercial and recreational importance (*Lates calcarifer, Chanos chanos, Megalops cyprinoides, Elops hawaiensis*), which commonly utilise off-channel habitats during early life-history stages [15], [17], [24]. To identify general patterns of wetland utilisation we examined parallel dynamics of catch per unit effort (CPUE) and size class at a species or genus level (where identification to species level was not possible) over three annual cycles. Plotted together as a time series, CPUE and modal size-class data enabled the examination of demographic trajectories and shifts within the populations of taxa through time. These dynamics in turn allowed interpretation of the processes underpinning wetland utilisation patterns. Such processes included recruitment (defined here as an annual population peak, dominated by the smallest recorded size class for that cycle), growth, mortality and emigration. Similar methods have previously been applied to identify functional groups within the estuarine fish assemblage [19].

CPUE was calculated as an average abundance over the 20 replicate pools in each month. For larger pools requiring multiple net hauls, only data from the net containing the greatest abundance of a species was used. Since certain individuals within a pool may have been released and recaptured in subsequent hauls, taking the net of greatest abundance ensured individuals were not accounted for more than once. Monthly CPUE and associated error structure were plotted against modal size-class trends for each taxon. Modal size classes were extracted from monthly plots of size-class distribution, fitted with a generalised additive model (GAM), for which the specified degrees of freedom were adjusted based on the size-class range. Where GAM curves were bimodal, two modal size classes were extracted for a single month. Size-class distributions were based on the sum of each 10 mm size-class increment across the 20 pools in each month (with the net of greatest abundance taken to represent each increment in the larger pools). *Stolephorus* spp. and *Acanthopagrus* spp. data were only resolved to the genus level due to difficulties distinguishing between early life-stages of species in the field. Laboratory identified specimens of *Stolephorus* were mostly *S. comersonii* and *S. brachycephalus*, while *Acanthopagrus* spp. was primarily composed of *A. australis*, and to a lesser extent *A. pacificus*.

For species sufficiently abundant in both the wetland and regional estuaries, CPUE vs. size-structure plots were qualitatively compared. Any large-scale disparities between the plots were considered as different utilisation patterns.

## Results

### Physical Data

**Figure 2:**
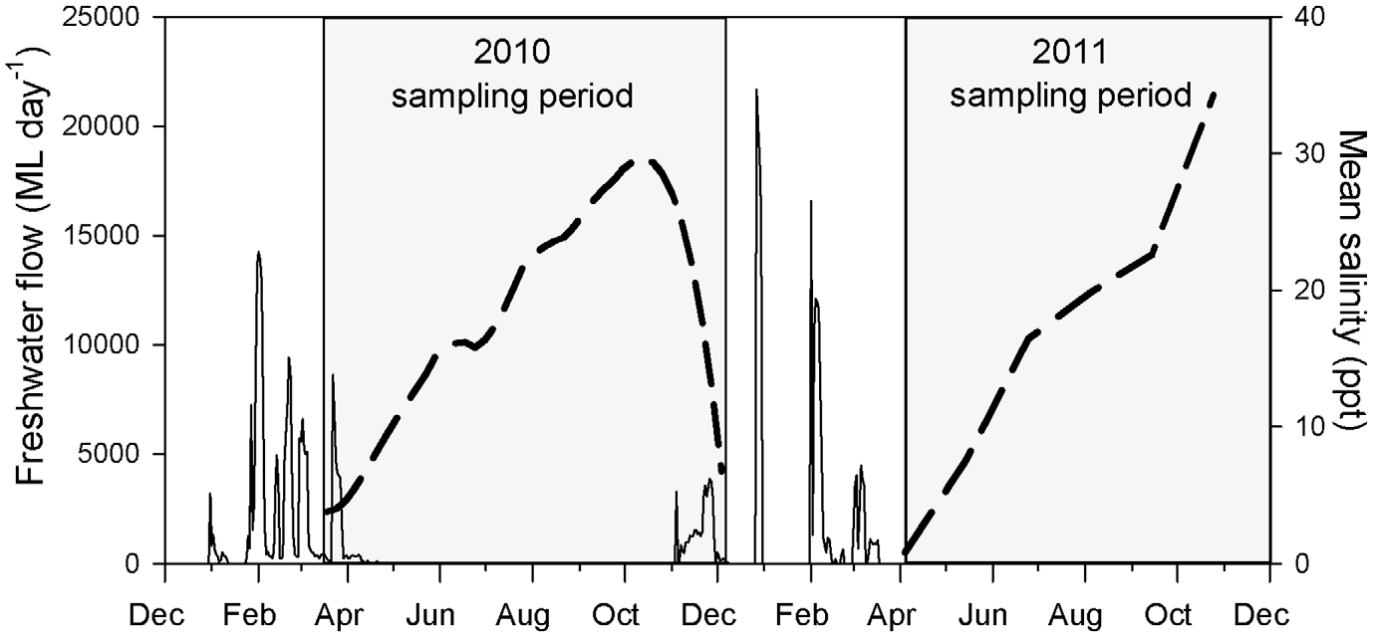
Freshwater flow and salinity changes in Annandale Wetland. Freshwater flowing over Aplin’s Weir (solid line plot) from December 2009 to December 2011 (measured as mega-litres per day), and the resulting salinity changes (smoothed with a Loess) in Annandale Wetland (dashed line) during the sampling periods (grey boxes) of 2010 and 2011. Freshwater flow data was provided by North Queensland Water. However, due to gauge-failure data were unavailable for much of December 2010 and all of January 2011, although the weir was flowing throughout these months.

Salinity in the wetland responded negatively to freshwater flow, ranging from 0–4 ppt directly after the wet season to >30 ppt pre-wet season (Fig. 2). Visibility was also loosely correlated with freshwater flow and water temperature displayed seasonal variation, yet these two variables provided little explanation of fish dynamics.

### Patterns of Fish Utilisation

**Figure 3:**
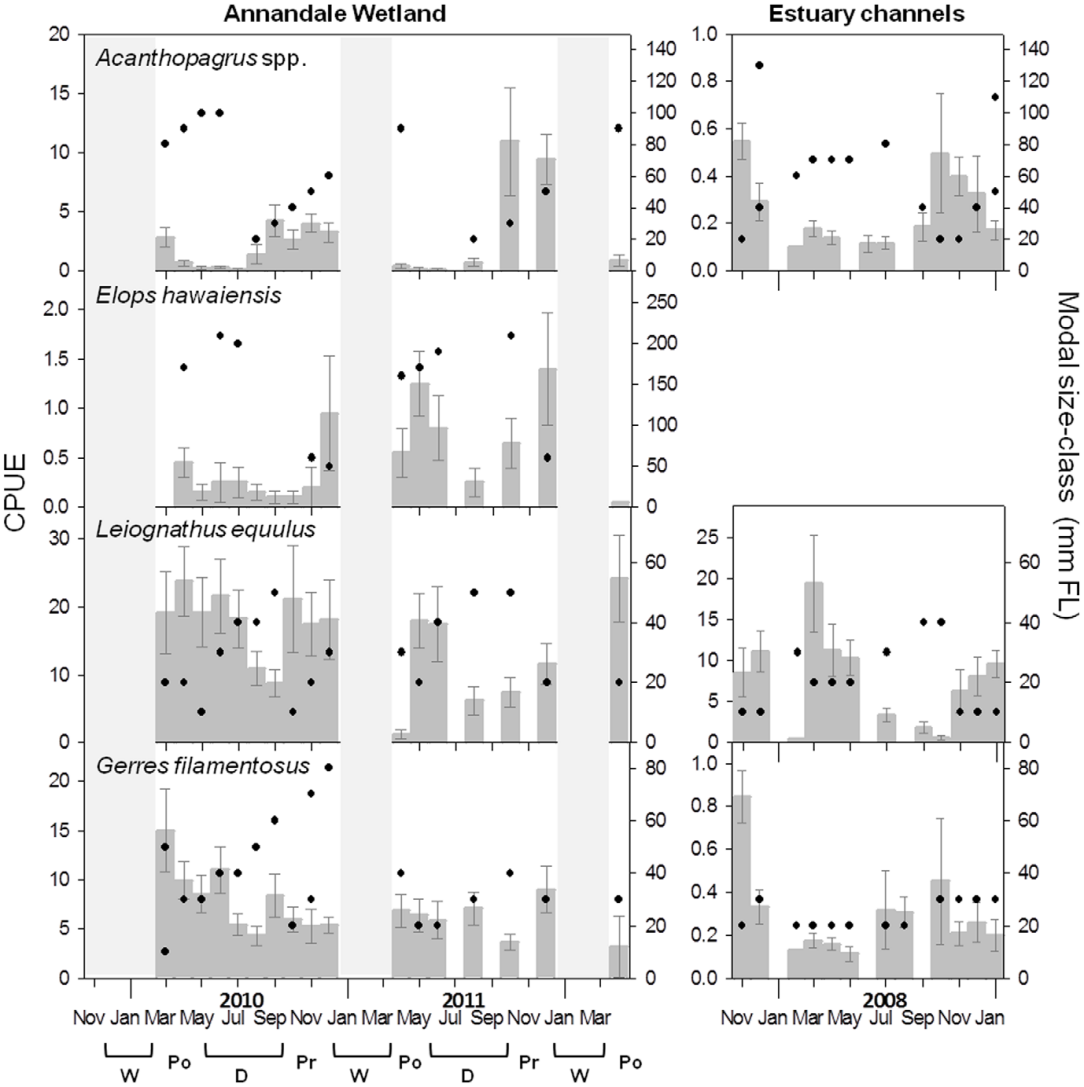
CPUE and modal size-class dynamics for taxa exhibiting patterns of classic nursery utilisation (CNU). Profiles of CPUE (±1 S.E.) (darker grey bars) averaged over the 20 pools in Annandale Wetland from March 2010–April 2012, matched with modal-size classes (filled black circles; measured as fork length (FL)). Where size-distributions were bimodal, two modes (black circles) are displayed for the same month. Sampling hiatuses are shaded in light grey, and generally represent periods when the salt-marsh surface was flooded with freshwater. Seasons have been labelled below the x axis (W = wet; Po = post-wet; D = dry; Pr = pre-wet). No data were collected in July, September, and November of 2011. Equivalent data are displayed for CPUE (±1 S.E.) and modal-size class averaged over the main bodies of 9 estuaries in the North Queensland region, over an extended annual cycle from pre-wet season 2007 to the 2008/2009 wet season. *Elops hawaiensis* was not caught in sufficient abundance in the 9 regional estuaries to display temporal dynamics.

**Figure 4:**
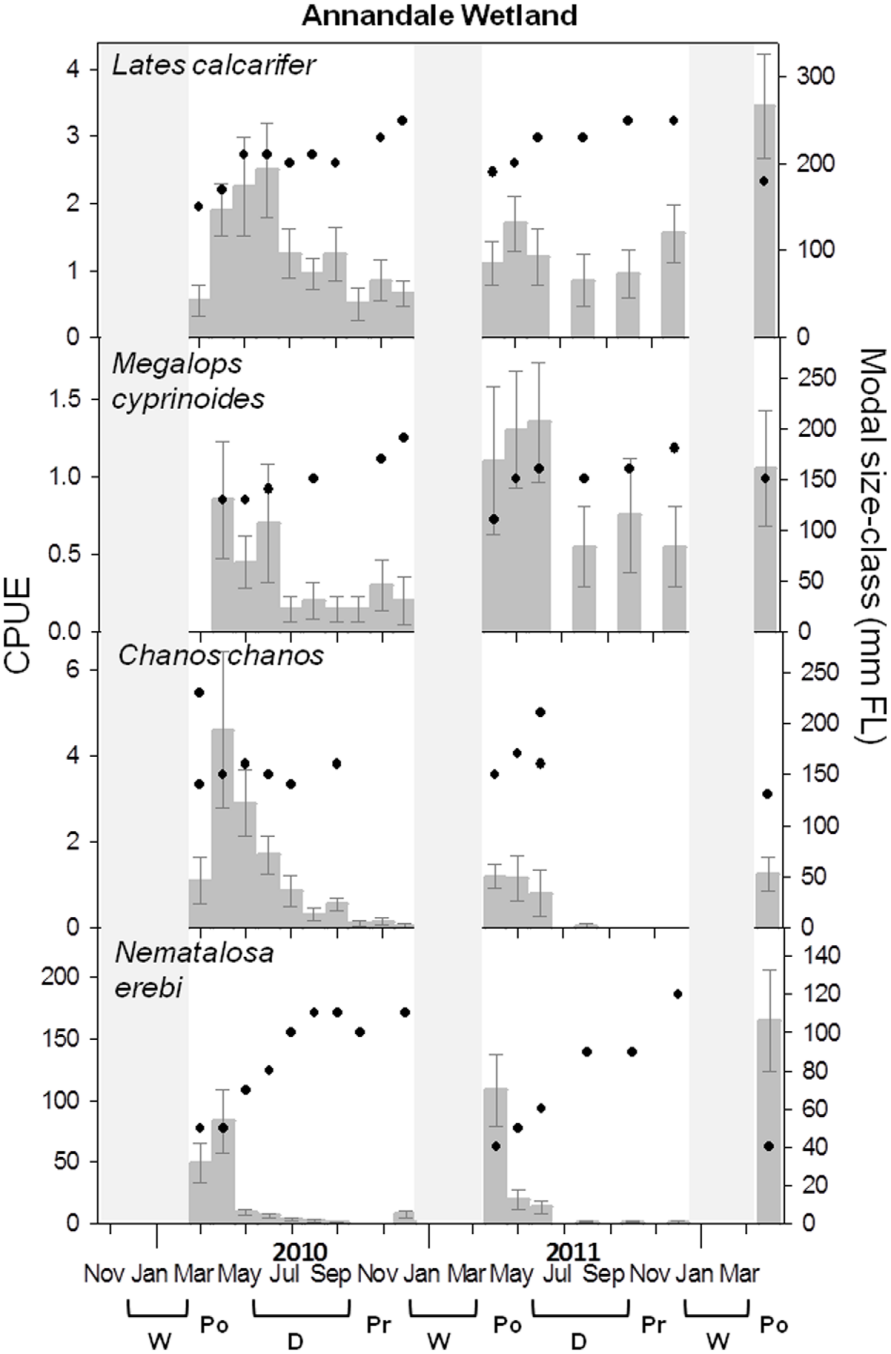
CPUE and modal size-class dynamics for taxa exhibiting patterns of delayed recruitment (DR). Details as per Figure 3. These taxa were not caught in sufficient abundance in the 9 regional estuaries to display temporal dynamics.

**Figure 5:**
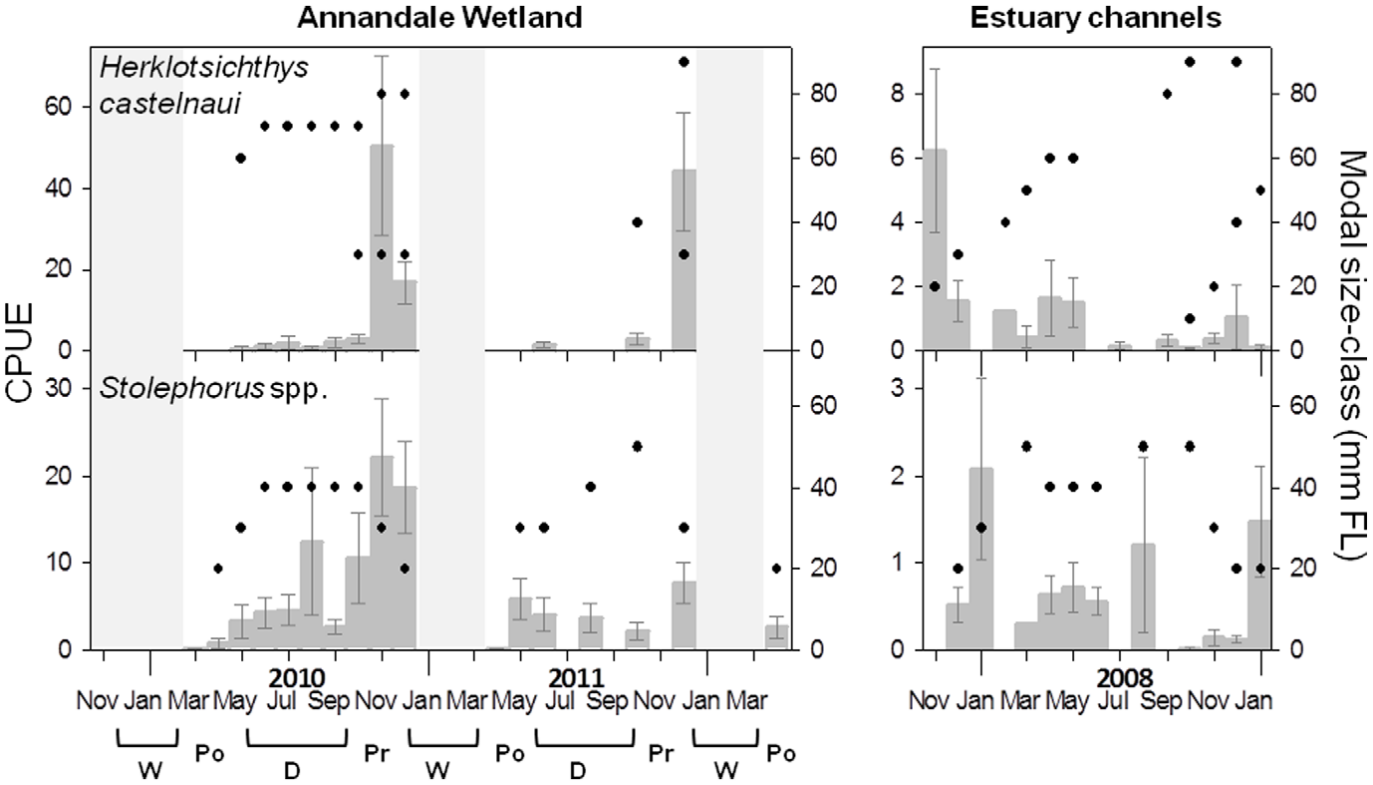
CPUE and modal size-class dynamics for taxa exhibiting patterns of interrupted persistence (IP). Details as per Figure 3.

**Figure 6:**
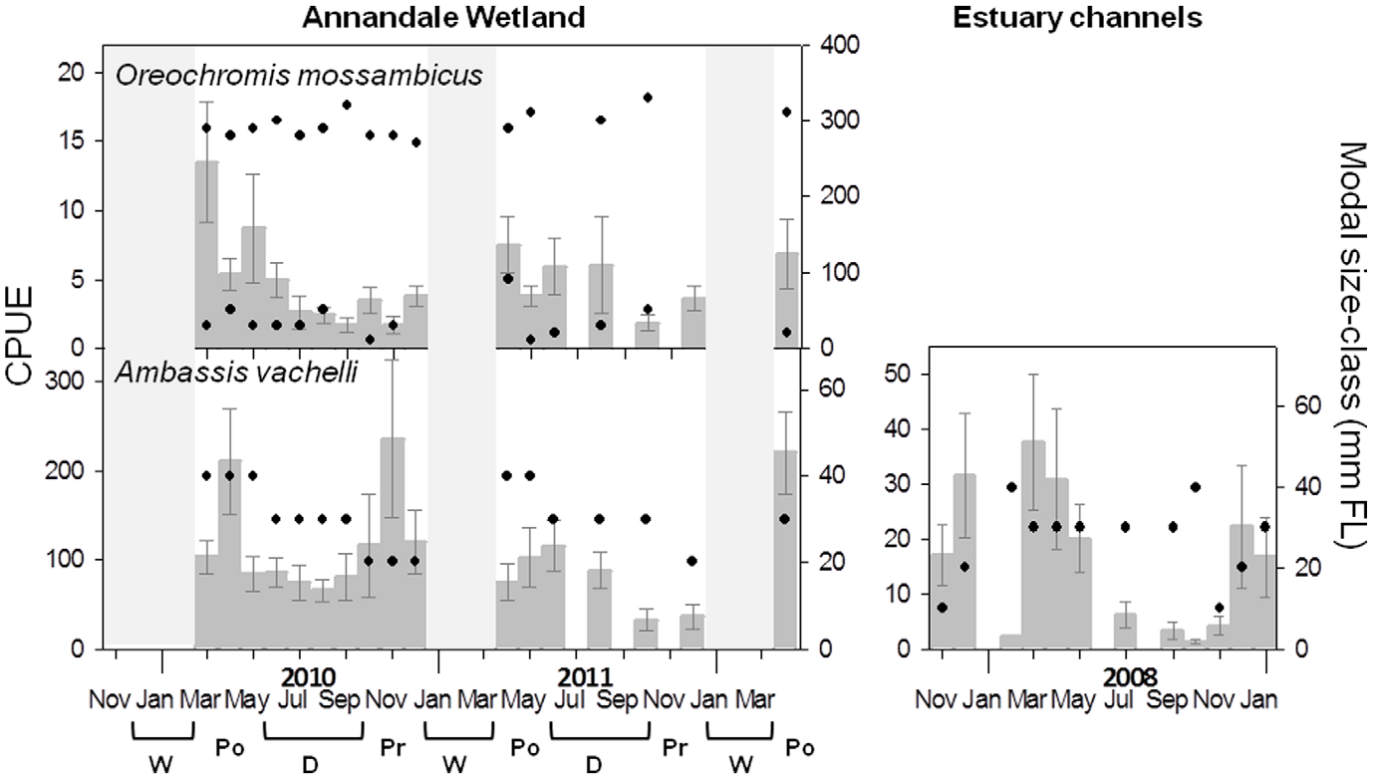
CPUE and modal size-class dynamics for taxa exhibiting patterns of facultative wetland residence (FWR). Details as per Figure 3. *Oreochromis mossambicus* was not caught in sufficient abundance in the 9 regional estuaries to display temporal dynamics.

Sampling produced 101 fish taxa, with 33 taxa collectively constituting 99.2% of the total catch (see Appendix S1). There were 10 dominant taxa, however two of these were small-bodied species (*Pseudomugil signifer* and *Hypseleotris compressa*) which were unsuitable for analysis since they were below gear selection size for substantial proportions of their life-cycles. The remaining 8 taxa (*Ambassis vachelli, Leiognathus equulus, Nematalosa erebi, Gerres filamentosus, Stolephorus* spp., *Herklotsichthys castelnaui, O. mossambicus* and *Acanthopagrus* spp.), together with the four commercial/recreational species, can be categorised into four groups based on CPUE vs. modal size-class plots (Figs. 3, 4, 5, 6): (i) Classic nursery utilisation, (ii) Delayed recruitment, (iii) Interrupted persistence, and (iv) Facultative wetland residence. These groups represent the dominant temporal utilisation patterns for the wetland, independent of taxonomic or life-history identities.

#### Classic nursery utilization

Four taxa (*L. equulus, Acanthopagrus* spp., *Elops hawaiensis,* and *G. filamentosus*) displayed a pattern of classic nursery utilisation (CNU), typified by cycles of recruitment at small size classes, followed by growth and then emigration. Taxa in the CNU group recruited as larvae and postlarvae (Fig. 3; Table 1), illustrated by heightened CPUE’s dominated by small size classes during peak recruitment periods. The timing and duration of recruitment varied between taxa. *Acanthopagrus* spp. and *E. hawaiensis* (Fig. 3) had relatively discrete recruitment periods, occurring August-September and November-December respectively, as illustrated by the progressive increase in modal size from the time of first recruitment, mirrored by simultaneous declines in abundance. Other CNU species displayed extended recruitment. For these, growth trajectories were periodically masked by the extended dominance of smaller size classes, suggesting numerous recruitment-growth cycles staggered over several months. *L. equulus* had an extended summer recruitment period, illustrated by the dominance of 10–30 mm FL size classes during the pre- and post-wet season. However, due to the sampling hiatus it remains uncertain whether this recruitment continued through the wet season itself. *G. filamentosus* also demonstrated an extended summer recruitment in 2010, but in 2011 displayed year-round recruitment, illustrated by year round dominance of 20–40 mm FL size classes.

**Table 1:** Approximate body lengths at important life-history landmarks for taxa recruiting to the wetland at small size classes (<40 mm FL), to determine how wetland utilisation patterns relate to life-histories.

Taxa	Length @ settlement	Reference	Common adult length
*Leiognathus equulus*	15 mm	[56]	200 mm TL
*Acanthopagrus* spp.	14 mm	[57]	300 mm TL
*Elops hawaiensis*	35 mm	[58]	500 mm SL
*Gerres filamentosus*	10 mm	[59]	150 mm SL
*Stolephorus* spp.	23–27 mm	[60]	85 mm SL
*Herklotsichthys castelnaui*	21–33 mm	[61]	140 mm SL
*Ambassis vachelli*	10 mm	[62]	60 mm SL
*Oreochromis mossambicus*	–	–	350 mm TL

Length at settlement from planktonic to demersal forms is displayed, for pelagic species this is assumed from the length of larval-juvenile morphological transformation. This information allows developmental stage of recruitment to be interpreted. Common adult lengths follow FishBase [55] (TL = total length; SL = standard length).

CNU taxa displayed similar patterns between Annandale Wetland pools and estuary channels (Fig. 3). These taxa displayed no apparent response to wet season floods (Fig. 2); CPUE’s and modal size classes directly after floods followed regular cycles of recruitment, growth and emigration (i.e. no sharp decreases or increases were observed directly after the wet season).

#### Delayed recruitment

Three species (*L. calcarifer, M. cyprinoides, C. chanos*) were caught exclusively as advanced juveniles (i.e. beyond postlarvae; all modal sizes were >100 mm FL) (Fig. 4), despite sampling overlapping with spawning seasons (spanning pre-wet season to the end of the wet season (Table 2)). These species comprise the delayed recruitment (DR) group. In the present study the smallest recorded size classes dominated annual population peaks during post-wet season months. Whether this represents discrete post-wet season recruitment is unclear, since we cannot account for potential recruitment during the wet season sampling hiatus. However in 2010 it was evident that the bulk of recruitment of these species was delayed until the second month of sampling in April (Fig. 4). In contrast, *N. erebi* CPUE was relatively high from first sampling in March (Fig. 4), and despite the smallest recorded size classes being 40–50 mm FL (representing advanced juveniles; Table 2), observed patterns are likely to represent the tail of a wet season recruitment dominated by smaller size classes. For each of these species, recruitment was followed by a maturation period where modal size increased as abundances declined through the year. However, *C. chanos* and *N. erebi* persisted for shorter periods than the other two species in this group.

**Table 2:** Early life history parameters of species only caught at advances sizes.

Species	Spawning period	Reference	Size in March-April(mm)	Reference	Size @ 1 year(mm)	Reference
*Lates calcarifer*	Nov-Mar; Oct-Feb	[17], [63]	∼200	[64]	∼300	[63], [64]
*Chanos chanos*	Nov-Mar	[31]	–	–	>150	[65]
*Megalops cyprinoides*	Oct-Feb	[15], [17]	∼100	[33]	–	–
*Nematalosa erebi*	Little seasonality; peaks early in wet	[66]	–	–	∼100	[67]

Spawning periods for widespread species refer to knowledge of periodicity in the tropics. Sizes in March-April are only considered for tropical Australian estuaries and refer to post-wet season sizes. This information is necessary to gauge the developmental stage of these delayed recruiting species.

#### Interrupted persistence

Two taxa (*H. castelnaui* and *Stolephorus* spp.) recruited as larvae or post-larvae in the pre-wet season (the interrupted persistence (IP) group), illustrated by large peaks in CPUE dominated by size classes of 20–30 mm FL (Table 1) in November-December (Fig. 5), followed by a complete absence directly after wet season freshwater flows (Fig. 2), with varying extents of re-colonisation of larger size classes (60–80 mm FL) post-wet to early dry season. This trend contrasted with more consistent patterns of CPUE in estuary channels (Fig. 5).

#### Facultative wetland residents

Two species (*A. vachelli* and *O. mossambicus*) displayed fluctuating CPUE’s through the year that matched with consistent size structures (facultative wetland resident (FWR) group). These trends reflected year-round occurrence of early post-settlement stages (represented by modal size classes of 20–30 mm FL for *A. vachelli*; and <90 mm FL for *O. Mossambicus* (Table 1)), in addition to larger juveniles and adults (Fig. 6). The simultaneous presence of both juveniles and adults is evident in the discrete bimodal size structure of *O. mossambicus* populations, represented by consistent occurrence of modal sizes of ∼300 mm FL in addition to modal sizes <90 mm FL (Fig. 6; Table 1). Although not evident from the figure, *A. vachelli* was also present as adults year round, with consistent presence of 50 mm FL size classes (Table 1). Furthermore, *A. vachelli* exhibited similar trends of fluctuating abundance and constant size-structure in estuary channels (Fig. 6), while *O. mossambicus* was absent in samples from those channels.

## Discussion

There were diverse patterns of utilisation among the 12 taxa analysed, defined by taxa-to-taxa differences in the details of CPUE and size-structure dynamics. Despite differences in detail these taxa could be broadly categorised into four groups based on similar patterns of wetland usage. Most taxa demonstrated a surprising tolerance to the severe and abrupt shifts in salinity, although for many taxa, utilisation patterns were strongly modified by other effects of freshwater flow. In general, utilisation patterns reflected the relationship of life-history schedules, physical tolerances, and habitat requirements with variations in hydrological connectivity, physical conditions, and habitat availability mediated by the interplay of tidal and freshwater flow.

### Patterns of Utilisation

Four taxa (*L. equulus, Acanthopagrus* spp., *G. filamentosus,* and *E. hawaiensis*) display CNU patterns, following cycles of post-larval recruitment, growth, and assumed emigration to other habitats upon reaching critical juvenile sizes [25]. This pattern has previously been described by Robertson & Duke [19] for fish use of a tropical Australian estuary. The uninterrupted nursery dynamics and mutuality of pattern between Annandale Wetland and estuary channels in the region, suggest that CNU taxa are tolerant of the abrupt marine-freshwater shifts experienced in estuarine pools, and are simply using the wetland as they would the estuary channel. The possible exception is *E. hawaiensis,* which has not been captured in abundance in previous studies sampling estuary channels across numerous systems in the region [19], [26].

Two taxa, *H. castelnaui* and *Stolephorus* spp., display classic nursery ground dynamics in estuary channels, but in Annandale wetland, utilisation was interrupted by the advent of the wet season. Although estuary channel data were averaged over the full length of the estuary, details of the distribution of these species suggest they move downstream after freshwater flow events [20]. These are plantkivorous fish, so it is likely that freshwater flows push food aggregation zones further downstream [27]. Studies in temperate estuaries have attributed aggregations of planktivorous fish to the accumulation of plankton around the maximum turbidity zone (MTZ) [28]. MTZ’s form at the fresh-saltwater interface of estuaries [29], and are spatially variable, shifting downstream during periods of high freshwater input. Consequently, restricted wetland utilisation by these planktivorous species probably reflects occupation limited to periods when conditions are suitable for them or when their food source is present.

Four species (*L. calcarifer, M. cyprinoides, N. erebi,* and *C. chanos*) display a delayed recruitment to the wetland, arriving at advanced-size juvenile stages during wet or post-wet season months. Consequently, it is implicit that these species initially settle as post-larvae elsewhere. For *N. erebi,* settlement occurs in permanent freshwater reaches (e.g. above Aplin’s Weir), due to exclusive freshwater spawning [30]. While it is possible that *N. erebi* recruited as early post-settlement juveniles during the wet season sampling hiatus, recruitment to tidal wetlands is essentially decoupled from life-history schedule, and the exact size at recruitment is dependent on the relationship between timing of spawning and the timing of freshwater flows, which allow movement to the wetland. The other three species (*L. calcarifer*, *C. chanos,* and *M. cyprinoides*) spawn in coastal marine waters [17], [31], [32]. While little is known of the early life-history of *M. cyprinoides* and *C. chanos, L. calcarifer* has a complex early-life history linking multiple coastal habitats. *L. calcarifer* and *M. cyprinoides* post-larvae recruit to shallow habitats associated with elevated wet season water levels, including supra-littoral depressions on saltpans and ephemeral freshwater and brackish swamps [14], [17]. Recruitment of advanced juvenile *L. calcarifer* into subtidal estuarine habitats synchronises with draw-down of these ephemeral habitats at the end of the wet season [18]. Meanwhile juvenile *M. cyprinoides* migrate upstream during post-wet season months [33], [34]. The delayed patterns of recruitment in the present study suggest that a similar habitat progression may occur in the Ross River, with recruiting individuals having previously occupied flooded ephemeral wetlands earlier in the wet season. This ephemeral wetland could potentially be the seasonally flooded areas of salt-marsh surrounding the pools on Annandale Wetland.

Following recruitment, *L. calcarifer* and *M. cyprinoides* persist and grow on the wetland through the year, yet persistence of *N. erebi* and *C. chanos* is particularly brief, with an absence or negligible abundance from post-wet season to early dry season. Brief persistence may be the result of migration, or mortality without ability for re-colonisation. Falling water levels during this period could cause *N. erebi* to migrate to preferred deeper waters [35] or expose them to elevated predation from both avian [36] and piscine predators. *L. calcarifer* is a major predator of *N. erebi* [37] and recruits to the wetland during this period. Furthermore, despite the capability of *N. erebi* to persist when captive in hypersaline lakes [38], increasing salinities may cause sub-lethal stress and trigger emigration to other habitats. *C. chanos* on the other hand is an active roving fish, and may be restricted by the volume of the pools as water levels drop in the post-wet season [24], prompting emigration.

In contrast to the nursery-orientated utilisation of the rest of the assemblage, two facultative wetland residents (FWR) (*A. vachelli* and *O. mossambicus*) were present in the wetland year-round both as young juveniles and adults. Continual presence of young juveniles suggests spawning may occur within the wetland or perhaps adjacent habitats. For *A. vachelli* these trends occur at the scale of the entire estuary (this study and [39]), and recruitment may reflect both colonisation from the estuary channel and spawning within the wetland. *O. mossambicus* however is generally considered a freshwater-spawning species and appears to primarily recruit to Annandale Wetland from freshwater reaches during the wet season. However, the surprising resilience in the number of both adults and juveniles through the year (despite removal upon capture) is indicative of re-colonisation from adjacent estuarine habitats, and subsequent spawning in the wetland. The shallow, sheltered nature and soft sediment common in the wetland appears to provide ideal habitat for the formation of breeding arenas (circular depressions in the sediment called ‘Leks’) [40], which were frequently observed in wetland pools during the sampling period (pers. obs). Studies of *O. mossambicus* in similar tropical estuaries suggest they are capable of spawning in seawater salinities, but distributions are limited to torpid waters in the upper estuary or enclosed water bodies [41].

### Linking Pattern and Process

Estuarine floodplain wetlands are essentially satellite habitats. With the exception of the two facultative wetland residents, which are possibly capable of self-recruitment and resilient to the prolonged periods of isolation often experienced in lesser-connected wetland units [42], the majority of taxa use estuarine pools exclusively as juveniles and are dependant on connectivity to other habitats. The large contribution of juveniles dependant on connectivity to other habitats probably explains why Sheaves & Johnston [8] found that re-colonisation based factors were more important than local factors in driving fish assemblages of sub-tropical pools. The main source of recruits for estuarine pools is the estuary channel, for which the assemblage itself is governed by multiple processes influencing different faunal components [20]. However, from the perspective of fringing habitats the estuary channel can simply be perceived as source from which recruits are drawn.

The nature of connection between estuary channels and floodplain wetlands will play a large role in structuring the wetland assemblage. For the members of the CNU group, which use pools indiscriminately as just another estuarine habitat, the regime (i.e. frequency and timing) and physical integrity (i.e. depth and presence of physical barriers) of connections to the estuary channel are likely to be the sole regulators of wetland utilisation pattern. In Annandale Wetland estuary channel-to-pool connections were established through most tidal cycles, and utilisation of several taxa mirrored patterns in the estuary channel. However, in reality regimes of estuary connection across estuarine floodplains are highly variable from wetland to wetland, occurring on scales of days, weeks, months, and sometimes years [8]. This variety of connection regime among floodplain wetlands is likely to result in spatio-temporal asymmetries in assemblage compositions, through matching and mismatching of connection events with the availability of different taxa to recruit, particularly larval and post-larval stages which are highly abundant for short windows [43]. However, this effect may be tempered somewhat by the general overlapping of spawning and recruitment with elevated wet season water levels, which may enable many estuarine taxa to access floodplain wetlands that would otherwise be inaccessible via tidal connections alone.

Beyond the simple effect of enhancing connection depths and durations, other effects of wet season freshwater flows appear to modify wetland utilisation patterns and assemblage structures. Flows move certain planktivorous species (IP group) out of the wetland system, and simultaneously donate many *N. erebi* and *O. mossambicus* from permanent freshwater sources. Meanwhile, the extent of freshwater flooding will regulate use of ephemeral wetlands that certain members of the DR group initially recruit to. Effective use of these intermediate habitats is likely to modify the extent, timing, and size of recruitment of these larger and mostly predatory species (*L. calcarifer* and *M. cyprinoides*) to estuarine pools.

Despite the presence of Aplin’s Weir directly upstream of the study site, the wet season flow dynamics observed in study are similar to dynamics in unregulated river systems [44]. In unregulated river systems however, weaker rainfall is more likely to initiate stream flow [44], and freshwater spawned species will have the potential to repopulate tidal wetlands more frequently through the year. However, there are few rivers on Australia’s North East coast without weirs or dams [45], and so the physical and biological patterns observed in this study are likely to be representative of the functioning of estuarine systems in the region.

The pivotal role of freshwater flow in mediating key physical and biological processes of estuarine floodplain wetlands adds a profound layer of variability to wetland functioning since wet season rainfall in dry tropical and sub-tropical regions is inter-annually inconsistent, following a loose cycle of wet and dry climactic periods spanning multiple years, largely associated with ENSO cycle [46]. Extended periods of negligible freshwater flow into dry- tropical and sub-tropical estuaries are not uncommon [44], and reliability of flow is expected to become increasingly erratic with climate change [47], a phenomenon exacerbated by the widespread regulation of river systems [45]. Further work is required during dry climactic periods to uncover the full influence of flow denial on wetland utilisation patterns. The response of the DR group to a drought period is of particular interest, since the use of intermediate habitats (i.e. seasonally flooded lowlands) will be disabled [48]. In addition, a clearer understanding of the ontogenetic sequence of habitat use for these species’ is required to fully understand the processes regulating nursery function.

Due to their diversity of form and connectivity, several additional processes operating at finer spatio-temporal and conceptual scales are likely to further complicate assemblage structure and dynamics of estuarine floodplain wetlands. This includes taxonomic and ontogenetic differences in locomotory capabilities [49], [50], movement-based behaviours [51], [52] and sub-habitat associations [23], [53]. Consequently, further work is required to establish the recruitment potential of the fish assemblage to wetlands of varying connectivity and morphology, through examining among-pool spatial patterns. Additionally, the potential homogenising effect of freshwater floods on floodplain pool assemblages needs to be explored [54].

This study demonstrates the diversity of utilisation pattern and complexity of associated drivers inherent in a coastal nursery habitat characterised by dynamic physical conditions and a high taxonomic diversity. It is evident that the processes regulating the occurrences of fish are not mutual across the assemblage, but vary among taxa, with different species responding differently to the same hydrological connectivity event. Therefore any future change in hydrological regime in this system, driven by natural fluctuation, climate change or water regulation, will have differing impacts on different members of the assemblage. Consequently, the assemblage composition and ecological function of estuarine floodplain wetlands is prone to variation among years, and there is a need to incorporate the diversity of assemblage drivers into understandings of habitat function, conservation and management.

## Supporting Information

Appendix S1 Summary of catch - raw abundance across all 20 pools summed over the complete sampling period featuring taxa collectively constituting 99.2% of the total catch. Taxa which alone constitute >1% of the catch are highlighted in bold, and were selected for analysis, *with the exception of *Pseudomugil signifier* and *Hypseleotris compressa* whose small body sizes limited interpretation of size-structure dynamics under the applied techniques.
(DOC)
