# Supplementary material for: Fish Utilisation of Wetland Nurseries with Complex Hydrological Connectivity
Source: PLoS One. 2012 Nov 9;7(11):e49107. doi: 10.1371/journal.pone.0049107 (PMC3494659; doi:10.1371/journal.pone.0049107)
Supplement: Appendix S1 — Summary of catch - raw abundance across all 20 pools summed over the complete sampling period featuring taxa collectively constituting 99.2% of the total catch. Taxa which alone constitute >1% of the catch are highlighted in bold, and were selected for analysis, *with the exception of Pseudomugil signifier and Hypseleotris compressa whose small body sizes limited interpretation of size-structure dynamics under the applied techniques. (DOC) [file pone.0049107.s001.doc]

| Family  ***Appendix****:* Summary of catch - raw abundance across all 20 pools summed over the complete sampling period featuring taxa collectively constituting 99.2% of the total catch. Taxa which alone constitute >1% of the catch are highlighted in bold, and were selected for analysis, *with the exception of *Pseudomugil signifier* and *Hypseleotris compressa* whose small body sizes limited interpretation of size-structure dynamics under the applied techniques. | Species | Pool |  |  |  |  |  |  |  |  |  |  |  |  |  |  |  |  |  |  |  |
| --- | --- | --- | --- | --- | --- | --- | --- | --- | --- | --- | --- | --- | --- | --- | --- | --- | --- | --- | --- | --- | --- |
|  |  | A1 | A2 | A3 | A4 | A5 | B1 | B2 | B3 | B4 | B5 | B6 | B7 | B8 | C1 | C2 | C3 | C4 | C5 | C6 | C7 |
|  |  |  |  |  |  |  |  |  |  |  |  |  |  |  |  |  |  |  |  |  |  |
| Ambassidae | ***Ambassis vachelli*** | 2284 | 2170 | 916 | 849 | 39 | 2057 | 802 | 2207 | 1837 | 5278 | 153 | 3359 | 353 | 2198 | 3923 | 1141 | 4823 | 2169 | 2330 | 4304 |
| Apogonidae | *Glossamia aprion* | 8 | 3 | 1 | 3 | 1 | 3 |  | 4 | 1 | 9 |  | 4 | 17 | 2 |  | 1 |  |  | 3 |  |
| Atherinidae | *Craterocephalus stercusmuscarem* | 62 | 24 | 41 | 32 | 50 | 3 | 14 | 21 | 7 | 19 | 5 | 3 |  | 7 |  | 1 |  |  |  |  |
| Belonidae | *Stongylura kreffti* | 9 | 5 | 15 | 15 |  | 11 | 6 | 8 | 4 | 5 |  | 4 |  | 5 | 1 | 1 | 2 | 1 |  | 2 |
| Centropomidae | *Lates calcarifer* | 21 | 78 | 76 | 26 | 1 | 22 | 8 | 5 | 35 | 75 | 13 | 26 | 7 | 23 | 56 | 5 | 30 | 30 | 3 | 8 |
| Chanidae | *Chanos chanos* | 20 | 23 | 10 | 9 | 45 | 1 | 4 | 20 | 18 | 11 | 7 | 6 | 7 | 95 | 24 | 24 | 13 | 17 | 10 | 10 |
| Cichlidae | ***Oreochromis mossambicus*** | 35 | 125 | 142 | 67 | 76 | 69 | 6 | 30 | 86 | 161 | 149 | 46 | 147 | 132 | 101 | 169 | 75 | 48 | 105 | 104 |
| Clupeidae | ***Herklotsitchthys castelnaui*** | 453 | 221 | 392 | 169 | 4 | 466 | 28 | 60 | 1018 | 214 | 3 | 138 | 6 | 14 | 18 | 5 | 41 | 50 | 35 | 23 |
|  | ***Nematalosa erebi*** | 401 | 810 | 750 | 828 | 934 | 156 | 86 | 182 | 246 | 877 | 1164 | 700 | 280 | 1082 | 681 | 275 | 350 | 152 | 467 | 184 |
| Eleotridae | *Butis butis* | 7 | 2 |  | 3 | 1 | 8 | 4 | 6 | 9 | 4 |  | 4 |  |  | 4 | 2 | 2 | 4 | 3 | 10 |
|  | ***Hypseleotris compressa**** | 149 | 186 | 116 | 40 | 1447 | 151 | 245 | 60 | 189 | 79 | 809 | 58 | 425 | 158 | 90 | 514 | 6 | 11 |  | 17 |
| Elopidae | *Elops hawaiensis* | 14 | 18 | 14 | 5 | 12 | 5 |  | 3 | 7 | 15 | 26 | 7 | 3 | 12 | 3 | 6 | 2 | 3 | 4 | 3 |
| Engraulidae | ***Stolephorus* spp.** | 251 | 481 | 693 | 469 |  | 229 | 10 | 26 | 404 | 228 |  | 29 | 2 | 25 | 6 |  | 43 | 9 | 48 | 30 |
|  | *Thryssa hamiltonii* | 28 | 71 | 108 | 25 | 1 | 1 | 1 | 1 | 10 | 12 |  |  | 1 |  |  |  | 10 |  | 21 | 10 |
| Gerreidae | *Gerres erythrourus* | 37 |  |  | 1 | 1 | 20 | 4 | 7 | 9 | 9 |  | 7 | 1 | 3 | 16 |  | 1 |  | 1 | 1 |
|  | ***Gerres filamentosus*** | 183 | 211 | 276 | 114 | 15 | 484 | 159 | 258 | 222 | 195 | 2 | 96 | 20 | 159 | 141 | 43 | 82 | 73 | 138 | 278 |
| Gobiidae | Gobiidae sp. 1 | 95 | 48 | 83 | 38 |  | 28 | 1 | 14 | 100 | 44 |  | 17 | 48 | 1 | 3 | 1 | 8 | 29 | 96 | 131 |
|  | *Glossogobius circumspectus* | 49 | 13 | 9 | 9 | 1 | 164 | 107 | 48 | 112 | 82 |  | 8 | 3 | 4 | 17 | 2 | 9 | 24 | 44 | 53 |
|  | *Favinogobius reichei* | 14 | 1 | 25 |  |  | 8 | 12 | 9 | 15 | 11 | 2 | 3 | 10 | 4 |  | 15 |  | 1 | 1 |  |
| Hemiramphidae | *Zenarchopterus buffonis* | 1 |  |  |  | 1 |  | 5 | 1 |  | 2 |  |  |  |  |  |  | 14 | 17 | 1 | 26 |
| Leiognathidae | *Gazza minuta* |  |  | 15 |  |  | 31 |  |  | 63 |  |  | 2 |  |  |  |  | 15 |  | 1 | 16 |
|  | ***Leiognathus equulus*** | 335 | 358 | 622 | 298 |  | 1324 | 51 | 244 | 1483 | 1534 | 5 | 562 | 9 | 40 | 167 |  | 405 | 409 | 243 | 420 |
|  | *Secutor ruconius* | 8 | 12 | 14 |  |  | 10 |  | 11 | 14 | 26 |  | 8 |  |  |  |  | 11 |  | 3 | 8 |
| Lutjanidae | *Lutjanus argentimaculatus* |  |  |  | 1 |  | 11 | 5 | 3 | 2 | 1 |  |  |  | 5 | 2 | 1 | 7 | 3 | 4 | 14 |
| Megalopidae | *Megalops cyprinoides* | 1 | 38 | 47 | 15 | 21 | 2 |  |  |  | 2 | 23 | 5 | 1 | 8 | 11 | 4 | 3 | 4 | 7 | 21 |
| Monodactylidae | *Monodactylus argenteus* |  |  |  |  |  | 3 | 4 | 24 |  |  |  |  |  | 4 | 1 |  | 2 | 16 |  | 3 |
| Mugilidae | *Liza subvirids* | 15 | 5 | 16 | 3 | 3 | 8 | 13 | 15 | 8 | 26 | 22 | 10 | 21 | 42 | 21 | 53 | 14 | 7 | 29 | 37 |
|  | Mugilidae juv. | 16 | 8 |  |  | 14 | 1 |  | 1 | 1 | 15 | 154 | 1 | 82 | 42 | 2 | 102 | 1 | 2 | 10 | 24 |
|  | *Valamugil seheli* | 3 | 2 |  | 5 | 4 | 7 | 2 | 17 | 13 | 19 | 2 | 7 | 10 | 12 | 21 | 17 | 8 | 3 | 10 | 5 |
| Platycephalidae | *Platycephalus* juv. | 12 | 8 | 8 | 11 | 1 | 2 | 6 | 6 | 18 | 16 |  | 1 |  | 1 |  | 2 | 2 | 1 |  | 7 |
| Pseudomugilidae | ***Pseudomugil signifer**** | 8 | 4 | 1 | 1 | 3 | 46 | 47 | 209 | 37 | 101 | 142 | 19 | 87 | 226 | 7 | 54 | 1 | 1 | 1 | 16 |
| Scatophagidae | *Selenotoca multifasciata* | 10 | 1 | 6 | 3 | 22 | 7 | 15 | 11 | 8 | 5 | 77 | 4 | 13 | 36 | 11 | 11 | 4 | 9 | 12 | 6 |
| Sparidae | ***Acanthopagrus* spp.** | 92 | 20 | 12 | 14 |  | 204 | 54 | 42 | 192 | 77 |  | 74 | 10 | 19 | 91 | 13 | 25 | 21 | 55 | 51 |
| Tetraodontidae | *Arohtron reticularis* | 1 | 1 |  | 3 |  | 7 | 5 | 21 | 9 | 4 |  |  |  | 1 | 4 | 1 | 17 | 3 | 1 |  |
| Toxotidae | *Toxotes chatareus* | 4 |  |  |  | 3 | 5 | 7 | 15 | 3 | 3 |  |  |  | 5 | 1 | 3 | 3 | 1 | 3 | 14 |
